# Our Sesqui-Centennial

**Published:** 1880-11

**Authors:** 


### EDITORIAL DEPARTMENT.

"If thou hast truth to utter, speak;
And leave the rest to God."

Our Sesqui-Centennial.—Shall we attempt to indite an apostrophe to the time and the circumstances that conspired in founding the charming and delightful city in which we reside? Or, shall we note only a few of the grand results achieved in a century and a half, on the hills and vales that environ the head waters of the beautiful Chesapeake?

The space at our disposal would deny us, did the inclination prompt the venture of the former, and we must, therefore, restrict what we may be able to say, to a brief notice of the latter, as

"We live in deeds, not years; in thoughts, not breaths,
In feelings, not in figures on the dial:
We should count time by heart throbs, when they beat
For God, for man, for duty. He most lives
Who thinks most, feels the noblest, acts the best.
Life is but a means unto an end—that end
Beginning, mean, and all things—God."

From a little hamlet nestling close to the Patapsco's edge, and sending out some scattered dwellings into the adjacent hill-sides, with no water craft beyond a few fishing smacks or Indian canoes; our city abounds with palatial structures, has a population of near four hundred thousand souls, and a commerce whose sails now whiten every sea. This metamorphose or transformation has been wholly wrought within the century and a half just ended, and in commemoration of which, more than an entire week has been devoted to its celebration. To speak of this in detail would not comport well with the general character and scope of a Medical Journal, suffice it then to say, that if Lord Baltimore ever dreamed, or pictured in his imagination, that a large and prosperous town would grow out of his little hamlet, he would have beheld, could he have seen it, something far beyond this in the grand city, in its Sesqui-Centennial attire, appearing as though a magician's wand, and not the hand of man, had created the fairy scenes of the recent festival. Such was the magnificence and gorgeousness of display on every side, that visitors from all quarters declared that the like had never been seen before upon these western shores. It would be an useless expenditure of time, and consumption of valuable space, to revert, and recapitulate what has been said already in the pages of this journal, concerning the Medical and eleemosynary institutions existing, or in process of erection, in this city, as our readers have been made familiar with them all. We are enabled, however, to furnish them with extracts from papers that have been prepared by gentlemen of Baltimore, upon medical matters, which are calculated to interest them, in this and the succeeding issue of the journal.

